# Impact of the COVID-19 pandemic on emergency department visits: reference center

**DOI:** 10.31744/einstein_journal/2021AO6467

**Published:** 2021-08-09

**Authors:** José Leão de Souza, Vanessa Damazio Teich, Anna Carolina Batista Dantas, Daniel Tavares Malheiro, Marcio Aparecido de Oliveira, Eduardo Segalla de Mello, Miguel Cendoroglo

**Affiliations:** 1 Hospital Israelita Albert Einstein São PauloSP Brazil Hospital Israelita Albert Einstein , São Paulo , SP , Brazil .

**Keywords:** Coronavirus infections, COVID-19, SARS-CoV-2, Emergency medical services, Health economics

## Abstract

**Objective:**

To analyze the impact of COVID-19 on emergency department metrics at a large tertiary reference hospital in Brazil.

**Methods:**

A retrospective analysis of consecutive emergency department visits, from January 1, 2020, to November 21, 2020, was performed and compared to the corresponding time frame in 2018 and 2019. The volume of visits and patients’ demographic and clinic characteristics were compared. All medical conditions were included, except confirmed cases of COVID-19.

**Results:**

A total of 138,138 emergency department visits occurred during the study period, with a statistically significant (p<0.01) reduction by 52% compared to both 2018 and 2019. This decrease was more pronounced for pediatric visits – a drop by 71% in comparison to previous years. Regarding clinical presentation, there was a decrease of severe cases by 34.7% and 37.6%, whereas mild cases decreased by 55.2% and 56.2% when comparing 2020 to 2018 and 2019, respectively. A 30% fall in the total volume of hospital admission from emergency department patients was observed during the study period, but accompanied by a proportional increase in monthly admission rates since April 2020.

**Conclusion:**

The COVID-19 pandemic led to a 52% fall in attendance at our emergency department for other conditions, along with a proportional increase in hospital admission rates of COVID-19 patients. Healthcare providers should raise patient awareness not to delay seeking medical treatment of severe conditions that require care at the emergency department.

## INTRODUCTION

A few months after the outbreak of coronavirus disease 2019 (COVID-19) in Wuhan, China, Brazil has become one of the epicenters of the disease. ^( 1 )^ To decrease the risk of contamination and avoid overloading the healthcare system, local governments have issued social containment decrees. ^( 2 )^ Since March 24, 2020, with social restrictions recommended in São Paulo, the largest city in Brazil, non-essential services were closed or greatly restricted, including elective medical examinations, medical appointments, and surgeries.

These measures, along with the fear of contracting COVID-19, are presumed to have notably affected the number of non-coronavirus-related hospital and emergency department (ED) visits, ^( 3 )^ despite the biased behavior of our population, both in private and public healthcare, to overuse ED as a shortcut or substitute to elective clinical consultations. A report from 2015 showed 34% of ED visits could be treated in primary care. ^( 4 )^ Previous reports from the severe acute respiratory syndrome coronavirus (SARS-CoV) outbreak, back in 2002, documented reduced ED visits in Hong Kong and Taiwan by 50%. ^( 5 , 6 )^ More recently, data from the COVID-19 pandemic have reported a 27% to 32% decrease in surgical ED visits, ^( 7 )^ and an impressive 76% drop in pediatric admissions. ^( 8 )^

While the reasons for this phenomenon have not been understood yet, it can result in delayed diagnosis and care for severe conditions, such as acute coronary syndrome and stroke, which is likely to result in increased long-term morbidity and higher out-of-hospital deaths. ^( 9 , 10 )^ Evaluating how the pandemic affects hospital attendance is paramount for adjusting and reorganizing the emergency care workforce and facilities.

## OBJECTIVE

To analyze the epidemiological impact of COVID-19 pandemic on emergency department visits in a large tertiary private reference center in Brazil.

## METHODS

In this cross-sectional study, medical records from all consecutive patients presenting to our ED, between January 1 to November 21, 2020, were analyzed and compared to those admitted in the same time frame during 2018 and 2019. Our health care complex includes five emergency care units and a tertiary private referral hospital with 592 beds. All medical conditions were included, except those related to COVID-19, confirmed by the positive result of real-time reverse-transcription polymerase chain reaction (RT-PCR) assay of nasal and pharyngeal swab specimens, and categorized as B34.2 according to the International Classification of Disease and Related Health Problems, tenth edition (ICD-10).

Data collected from electronic medical records included the daily number of patients, age, sex, diagnoses, clinical severity and the need for hospital admission. The diagnoses were classified according to the ICD-10. The severity of ED presentation was assessed according to the Emergency Severity Index (ESI). Age was distributed in groups, and children and adolescents were classified as aged ≤16 years, since this is cut-off applied in our organization for allocation in Pediatrics. Since the senior population was at greater risk for COVID-19, the individuals were sub-categorized for each decade: from 60 to 69, from 70 to 79, and above 80 years old. Volume distribution was performed weekly, in compliance to epidemiological week, defined in a standardized manner starting on Sunday and ending on Saturday, because the first epidemiological week of the year ends, by definition, on the first Saturday of January.

The primary outcome of the study was to analyze the impact of the COVID-19 pandemic on the volume of ED visits in 2020, as compared to the previous 2 years. Secondary outcome was to evaluate the impact on the main medical specialties at ED, and hospital admission rates from ED patients.

Patient confidentiality was preserved by de-identifying medical records. The study was approved by the Ethics Committee of *Hospital Israelita Albert Einstein* (HIAE), CAAE: 37238020.2.0000.0071, protocol 4.276.727.

Continuous variables were expressed as means with standard deviations, medians, minimum and maximum values. We conducted a paired Student’s *t* test for annual comparisons, grouped by two as 2018-2019, 2018-2020, and 2019-2020. Categorical variables were summarized as counts and percentages and applied to the χ ^2^ test. We used the F-test of equality of variances for the temporal analysis of epidemiological week volume, and monthly admission rates. Statistical significance was set by convention at p<0.05. We used IBM (SPSS), version 23.0 (IBM Corp., Armonk, New York, USA), and R-software, version 3.5.3 (R Foundation for Statistical Computing, Vienna, Austria), for statistical analysis.

## RESULTS

A total of 138,138 ED visits occurred during the study period, with a reduction by 52.7% compared to 2019 (n=292,344) and 52.2% to 2018 (n=289,118), with a statistically significant difference (p<0.01). As shown in table 1 , during the study period in 2020, the mean age of patients was significantly older compared to both 2019 and 2018 (36.5 *versus* 31.7 *versus* 31.2 years, respectively; p<0.01). When analyzing age groups, the proportional distribution showed an important percentual decrease in patients younger than 16 years (22% *versus* 33% *versus* 33.7%) and an increase in those aged 60 to 69 (65% *versus* 56% *versus* 56%), respectively for 2020, 2019 and 2018. Regarding clinical severity upon presentation, the proportional distribution was affected with a significant increase in severe cases grouped as ESI 1 or 2 (8.8% *versus* 6.7% *versus* 6.5%), accompanied by a decrease in mild cases with ESI 4-5 (45.1% *versus* 48.7% *versus* 48.1%) in 2020 *versus* 2019 and 2018, respectively. When comparing the relative decrease of ED visits by ESI group, there was a lower decrease of ESI 1-2 visits (37.6% and 34.7%), whereas ESI 4-5 decreased by 56.2% and 55.2%, when comparing 2020 to 2019 and 2018, respectively.

**Table 1:** Characteristics of the population served between years

Characteristics	Years			p value

	2018	2019	2020	
ED visits per year	289,118	292,344	138,138	<0.01
Age	31.2±22.2	31.7±22.2	36.5±21.2	<0.01
Age group, n (%)				<0.01
≤16	97,497 (33.7)	96,632 (33)	29,905 (22)	
17-59	7,394 (2.6)	7,664 (2.6)	4,544 (3.3)	
60-69	160,879 (56)	163,648 (56)	90,091 (65)	
70-79	14,350 (4.9)	14,723 (5.0)	8,234 (5.9)	
≥80	8,998 (3.1)	9,677 (3.3)	5,364 (3.9)	
Sex, n (%)				<0.01
Female	156,426 (54.1)	157,404 (53.8)	74,742 (54.1)	
Male	132,667 (45.9)	134,931 (46.1)	63,385 (45.9)	
Unknown	25 (0.1)	9 (0.1)	11 (0.1)	
ESI, n (%)				<0.01
Severe (ESI 1-2)	18,727 (6.5)	19,611 (6.7)	12,230 (8.8)	
Moderate (ESI 3)	119,635 (41.4)	118,339 (40.5)	57,718 (41.8)	
Mild (ESI 4-5)	139,157 (48.1)	142,309 (48.7)	62,325 (45.1)	
Not informed	11,599 (4.0)	12,085 (4.1)	5,865 (4.2)	

Results expressed as n, mean±standard deviation or n (%).

ED: emergency department; ESI: Emergency Severity Index.

**Figure 1:**
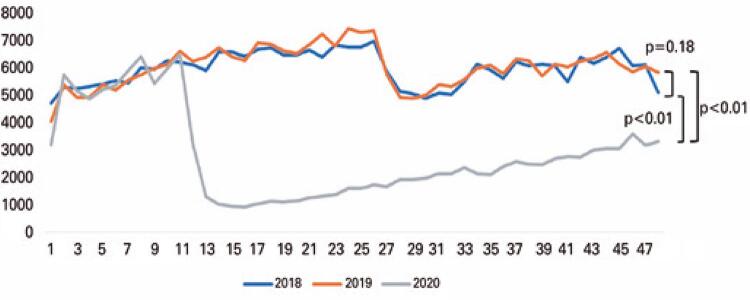
Volume of care in emergency care units distributed in epidemiological weeks (except COVID-19)

As of the 12 ^th^ epidemiological week in 2020, coinciding with the government-imposed measures for social containment, a weekly reduction in ED visits compared to the previous years could be seen, as shown in figure 1 . A mild recovery occurred after the 24 ^th^ week but remained statistically inferior in weekly volume when compared to both 2019 and 2018 (p<0.01).

**Figure 2:**
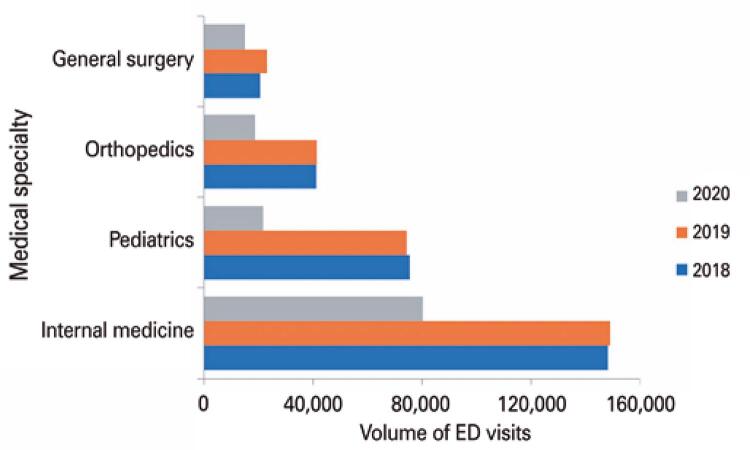
Volume of service according to medical specialties between the years

Following the patient profile change seen in table 1 , there was also a marked impact in our ED medical specialties volume, with a statistically significant decrease (p<0.01) in all during the study period, as seen in figure 2 . Markedly, pediatrics had it more pronounced, with a decrease by 70.7% and 71%, as compared to 2019 and 2018, respectively. Orthopedics decreased by 54.6% and 54.2%, internal medicine by 46.2% and 45.8%, and general surgery by 34.2% and 26.2%, in the same time frames.

ED: emergency department.

Comparing the proportionality of each medical specialty to total volume, while orthopedics had a slight decrease from 14.2% in the previous years to 13.6% in 2020, pediatrics had it more pronounced, with a decrease from 26.1% and 25.5% in 2018 and 2019, to 15.8% in 2020. Accordingly, internal medicine and general surgery increased in percentual volume, as seen in table 2 .

**Table 2:** Statistical comparison between the volume of care, according to medical specialties between years

Medical specialties	Years			p value

	2018 n=289,118	2019 n=292,344	2020 n=138,138	
Internal medicine	148,175 (51.2)	149,051 (50.9)	80,251 (58.1)	<0.01
Pediatrics	75,499 (26.1)	74,565 (25.5)	21,797 (15.8)	
Orthopedics	41,149 (14.2)	41,553 (14.2)	18,862 (13.6)	
General surgery	20,534 (7.1)	23,017 (7.9)	15,155 (10.9)	

Results expressed as n (%).

**Figure 3:**
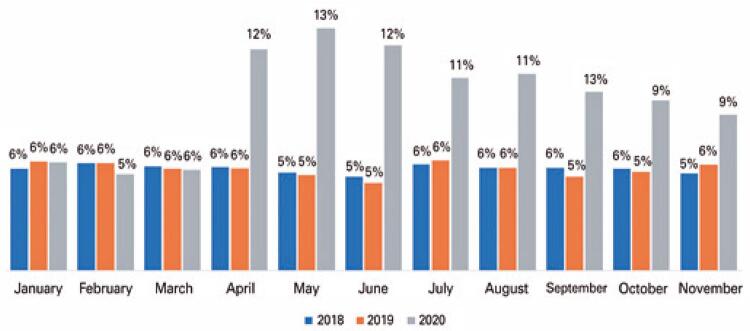
Monthly hospital admission rates from emergency department visits

The absolute number of hospital admissions from ED decreased by 31% in 2020, as compared to 2019, and by 30% to 2018. However, proportionally, when comparing the monthly number of hospital admissions originated from ED to its respective total volume of ED visits, there was a statistically significant (p<0.01) increase in admission rates as from April 2020, as shown in figure 3 .

## DISCUSSION

Our study shows evidence of a 52% reduction in the number of ED visits at our hospital in 2020, as compared to 2019 and 2018. Although much has been postulated about reasons for this phenomenon, such as fear of contamination or reduced likelihood of injury from workplace and traffic accidents, no published data has yet verified these explanations. Interestingly, it markedly declined in March 2020, after the 12 ^th^ epidemiological week, coinciding with the government-imposed measures for social containment.

Similar to our experience, other publications have reported reductions in non-COVID-19 related ED visits, since March 2020. ^( 11 , 12 )^ Data from the National Syndromic Surveillance Program (NSSP), in the United States, revealed a 42% decline in ED visits early in the pandemic, from March 29 to April 25, 2020. ^( 10 )^ In the Latin-American scenario, Pintado et al., showed a 79.9% decline in the number of patients treated at a Peruvian trauma reference center, comparing the months immediately pre- and post-lockdown. ^( 12 )^

Patient profile according to age groups varied during the pandemic. Contrary to our expectation of a proportional reduction of older adults, 65% of patients were aged 60 to 69 years, compared to 56% in the previous years. Montagnon et al., also found a similar increase in the proportion of elderly patients. ^( 13 )^ However, while we cannot have a particular reason for this, it can have resulted from the significant reduction in the proportion of patients younger than 16 years, from 33% and 22% in 2020.

Consonant to this reduction in volume of visits of younger patients, we also found a 71% decrease in pediatric consultations in 2020, compared to both 2019 and 2018. Early in the pandemic, Ciacchini et al. showed similar results in Italy, with a 76% reduction in pediatric ED admissions. ^( 8 )^ More recently, Nascimento et al., found a significant reduction from 81% to 45% in hospitalizations for respiratory diseases in children aged under 5 years. ^( 14 )^ As other studies have shown the impact of physical distancing and other lockdown strategies to slow down the spread of common respiratory viral diseases among children, ^( 15 )^ this reduction can also be accounted for an increase in education regarding the use of masks and hand hygiene, both by children and their parents.

Regarding disease severity, we found a relatively lower decrease in severe cases grouped in ESI 1 and 2, while there was a larger decrease in the volume of mild cases, grouped in ESI 4 and 5. Furthermore, a German study by Stöhr et al., reported a reduction in discretionary cardiovascular events, whereas severe conditions and unavoidable admissions, such as myocardial infarction and stroke, remained stable. ^( 16 )^ Yet to be proven with more consistent data, this larger decrease in mild clinical conditions at ED could be explained by the widespread use of telehealthcare during the COVID-19 pandemic. ^( 17 )^

Despite a 30% drop in the absolute number of hospital admissions from ED, we observed a proportional increase in admission rates since April 2020, as also demonstrated in other studies. ^( 18 )^ Although our data analysis could not prove this, we hypothesize this increase in admission rates can be due to a delay in seeking healthcare, leading to ED presentation of more severe clinical conditions. We suspect that fear of exposure to COVID-19 infection could have averted timely healthcare and thus impacted on clinical outcomes.

This study is limited due to its observational nature, since real causality for the observed decline in ED visits could not be determined. Moreover, as a single-center study, we cannot conclude it represents all regions of Brazil, for the impact of the COVID-19 pandemic has varied according to hospital characteristics and geographically across the country.

## CONCLUSION

The COVID-19 pandemic led to a significant decrease in our emergency department visits, especially in pediatrics, as from March 2020, right after government-imposed social containment measures. A proportional increase in hospital admission rates from emergency department was also observed during the study period. These findings suggest that healthcare providers should emphasize the importance of visiting the emergency department for severe conditions, which cannot be managed in other settings.
