# Supplementary material for: Impact of the COVID-19 pandemic on emergency department visits: reference center
Source: Einstein (Sao Paulo). 2021 Aug 9;19:eAO6467. doi: 10.31744/einstein_journal/2021AO6467 (PMC8354589; doi:10.31744/einstein_journal/2021AO6467)
Supplement: Translation

# Translation: Impacto da pandemia da COVID-19 no volume de atendimentos no pronto atendimento: experiência de um centro de referência no Brasil

**Authors:** José Leão de Souza, Vanessa Damazio Teich, Anna Carolina Batista Dantas, Daniel Tavares Malheiro, Marcio Aparecido de Oliveira, Eduardo Segalla de Mello, Miguel Cendoroglo

**Affiliations:** 1 Brazil Hospital Israelita Albert Einstein, São Paulo, SP, Brazil .

**Keywords:** Infecções por coronavírus, COVID-19, SARS-CoV-2, Serviços médicos de emergência, Economia da saúde

## Abstract

**Objetivo:**

Analisar o impacto da pandemia da COVID-19 nas métricas do pronto atendimento de um hospital terciário de referência no Brasil.

**Métodos:**

Uma análise retrospectiva das visitas consecutivas ao pronto atendimento, de 1^o^ de janeiro de 2020 a 21 de novembro de 2020, foi realizada e comparada ao mesmo intervalo nos anos de 2018 e 2019. O volume de atendimentos e as características clínicas e demográficas dos pacientes foram comparados. Todos os diagnósticos foram incluídos, exceto os casos confirmados de COVID-19.

**Resultados:**

Um total de 138.138 visitas ao pronto atendimento ocorreu durante o período do estudo, com redução estatisticamente significativa (p<0,01) de 52% do volume comparado tanto a 2018 como a 2019. Essa queda foi mais pronunciada nos atendimentos de pediatria, com redução de 71% se comparada aos números de anos anteriores. Em relação ao quadro clínico, houve redução dos casos graves em 34,7% e 37,6%, enquanto os casos leves caíram 55,2% e 56,2%, quando comparado 2020 a 2018 e a 2019, respectivamente. Uma queda de 30% foi vista no volume de admissões hospitalares originadas dessas visitas, porém houve aumento percentual da taxa de admissão mensal em relação ao volume desde abril de 2020.

**Conclusão:**

O impacto da pandemia da COVID-19 gerou redução de 52% no volume de atendimento do pronto atendimento por outras condições clínicas, bem como aumento proporcional na taxa de admissão hospitalar de pacientes com COVID-19. Os profissionais de saúde devem orientar seus pacientes a não atrasar a procura por atendimento médico de condições graves que precisem de cuidados no pronto atendimento.

## INTRODUÇÃO

Poucos meses após o surto da doença causada pelo coronavírus 2019 (COVID-19) em Wuhan, China, o Brasil se tornou um dos epicentros da doença.^( 1 )^Para diminuir o risco de contaminação e não sobrecarregar os sistemas de saúde, os governos municipais emitiram decretos de quarentena.^( 2 )^Desde 24 de março de 2020, com a instauração da quarentena em São Paulo, a maior cidade do Brasil, os serviços não essenciais foram encerrados ou fortemente restringidos, incluindo exames médicos eletivos, consultas e cirurgias.

Presume-se que essas medidas, junto do medo de contrair a COVID-19, tenham afetado notavelmente o número de atendimentos hospitalares e de pronto atendimento (PA) não relacionados ao coronavírus,^( 3 )^apesar do comportamento tendencioso de nossa população, tanto na rede privada quanto na saúde pública, para o uso excessivo do PA como atalho ou substituto para as consultas clínicas eletivas. Um relatório de 2015 mostrou que 34% dos casos atendidos em PA poderiam ser tratados em Atenção Primária.^( 4 )^Relatórios anteriores referentes ao surto do coronavírus da síndrome respiratória aguda grave (SARS-CoV), ocorrido em 2002, documentavam redução de 50% nas visitas ao PA em Hong Kong e Taiwan.^( 5 , 6 )^Mais recentemente, os dados da pandemia da COVID-19 mostraram redução de 27% a 32% nos atendimentos cirúrgicos no PA^( 7 )^e queda impressionante de 76% nas admissões pediátricas.^( 8 )^

Embora as razões para esse fenômeno ainda não sejam compreendidas, isso pode resultar em atrasos no diagnóstico e no atendimento de condições graves, como síndrome coronariana aguda e acidente vascular cerebral, o que provavelmente resulta em aumento na morbidade em longo prazo e no número de óbitos extra-hospitalares.^( 9 , 10 )^É fundamental avaliar como a pandemia afeta o atendimento hospitalar para a adaptação e a reorganização da força de trabalho e das unidades de atendimento de emergência.

## OBJETIVO

Analisar o impacto epidemiológico da pandemia da COVID-19 nos atendimentos de pronto atendimento em um grande centro terciário de referência privado no Brasil.

## MÉTODOS

Neste estudo transversal, os prontuários médicos de todos os pacientes consecutivos que se apresentaram no PA entre 1º de janeiro de 2020 e 21 de novembro de 2020 foram analisados e comparados com aqueles admitidos no mesmo período durante 2018 e 2019. O complexo de saúde estudado inclui cinco unidades de PA e um hospital terciário de referência privado, com 592 leitos. Todas as enfermidades foram incluídas, exceto as relacionadas à COVID-19 confirmadas por resultado positivo do ensaio de reação em cadeia da polimerase seguida de transcriptase reversa em tempo real (RT-PCR) de espécimes de esfregaço nasal e faríngeo e categorizadas como B34.2, de acordo com a Classificação Internacional de Doenças e Problemas Relacionados à Saúde – décima edição (CID-10).

Os dados coletados do prontuário eletrônico do paciente incluíram número diário de pacientes, idade, sexo, diagnósticos, gravidade clínica e necessidade de internação hospitalar. Os diagnósticos foram classificados de acordo com a CID-10. A gravidade da apresentação no PA foi avaliada de acordo com o Índice de Gravidade de Emergência (ESI - *Emergency Severity Index* ). A idade foi distribuída em grupos, e as crianças e adolescentes foram classificados como tendo ≤16 anos, pois esse é o ponto de corte aplicado na instituição para alocação em atendimento pediátrico. Como a população idosa estava em maior risco para COVID-19, ela foi subcategorizada para cada década: de 60 a 69, de 70 a 79, e acima de 80 anos. A distribuição dos volumes foi realizada semanalmente, de acordo com a semana epidemiológica, definida de forma padronizada, com início no domingo e término no sábado, pois a primeira semana epidemiológica do ano termina, por definição, no primeiro sábado de janeiro.

O desfecho primário deste estudo foi analisar o impacto da pandemia da COVID-19 no volume de atendimentos no PA em 2020 em comparação com os dois anos anteriores. O desfecho secundário foi avaliar o impacto nas principais especialidades médicas no PA e as taxas de admissão hospitalar de pacientes a partir desse atendimento.

A confidencialidade dos pacientes foi preservada pela desidentificação dos prontuários médicos. O estudo foi aprovado pelo Comitê de Ética do Hospital Israelita Albert Einstein (HIAE), CAAE: 37238020.2.0000.0071, parecer 4.276.727.

As variáveis contínuas foram expressas como médias com desvio-padrão, medianas e valores mínimo e máximo. Realizou-se um teste *t Student* pareado para as comparações anuais, agrupadas a cada 2 anos como 2018-2019, 2018-2020 e 2019-2020. As variáveis categóricas foram resumidas como contagens e percentagens e aplicadas ao teste do χ^2^. Utilizou-se o teste F de igualdade de variâncias para a análise temporal do volume epidemiológico semanal e das taxas mensais de internação. A significância estatística foi estabelecida como p<0,05. Foram usados os programas IBM (SPSS), versão 23.0 (IBM Corp., Armonk, Nova Iorque, Estados Unidos), e R, versão 3.5.3 (R Foundation for Statistical Computing, Viena, Áustria), para análise estatística.

## RESULTADOS

No período do estudo, ocorreram 138.138 atendimentos de PA, com redução de 52,7% em relação ao ano de 2019 (n=292.344) e de 52,2% em relação a 2018 (n=289.118), com diferença estatisticamente significativa (p<0,01). Conforme mostrado na tabela 1 , durante o período de estudo de 2020, a média de idade dos pacientes foi significativamente mais alta em comparação a 2019 e a 2018 (36,5 *versus* 31,7 *versus* 31,2 anos, respectivamente; p<0,01). Ao analisar as faixas etárias, a distribuição proporcional mostrou importante redução percentual nos menores de 16 anos (22% *versus* 33% *versus* 33,7%) e aumento nos de 60 a 69 anos (65% *versus* 56% *versus* 56%), respectivamente para 2020, 2019 e 2018. Em relação à gravidade clínica na apresentação, a distribuição proporcional foi afetada com aumento significativo nos casos graves agrupados como ESI 1 ou 2 (8,8% *versus* 6,7% *versus* 6,5%), acompanhada por diminuição nos casos leves com ESI 4-5 (45,1% *versus* 48,7% *versus* 48,1%) em 2020 em comparação com 2019 e 2018. Ao comparar a diminuição relativa de atendimentos no PA por grupo ESI, houve diminuição menor de visitas com ESI 1-2 (37,6% e 34,7%), ao passo que os atendimentos com ESI 4-5 diminuíram 56,2% e 55,2%, na comparação de 2020 com 2019 e com 2018, respectivamente.

**Tabela 1:** Características da população atendida entre os anos

Características	Anos			Valor de p

	2018	2019	2020	
Atendimentos no PA por ano	289.118	292.344	138.138	<0,01
Idade	31,2±22,2	31,7±22,2	36,5±21,2	<0,01
Faixa etária, n (%)				<0,01
≤16	97.497 (33,7)	96.632 (33)	29.905 (22)	
17-59	7.394 (2,6)	7.664 (2,6)	4.544 (3,3)	
60-69	160.879 (56)	163.648 (56)	90.091 (65)	
70-79	14.350 (4,9)	14.723 (5,0)	8.234 (5,9)	
≥80	8.998 (3,1)	9.677 (3,3)	5.364 (3,9)	
Sexo, n (%)				<0,01
Feminino	156.426 (54,1)	157.404 (53,8)	74.742 (54,1)	
Masculino	132.667 (45,9)	134.931 (46,1)	63.385 (45,9)	
Desconhecido	25 (0,1)	9 (0,1)	11 (0,1)	
ESI, n (%)				<0,01
Grave (ESI 1-2)	18.727 (6,5)	19.611 (6,7)	12.230 (8,8)	
Moderado (ESI 3)	119.635 (41,4)	118.339 (40,5)	57.718 (41,8)	
Leve (ESI 4-5)	139.157 (48,1)	142.309 (48,7)	62.325 (45,1)	
Não informado	11.599 (4,0)	12.085 (4,1)	5.865 (4,2)	

Resultados expressos por n, média±desvio-padrão ou n (%).

PA: pronto atendimento; ESI: Índice de Gravidade de Emergência.

A partir da 12ª semana epidemiológica de 2020, coincidindo com as medidas de contenção social impostas pelo governo, pôde-se verificar redução semanal de atendimentos no PA em relação aos anos anteriores, conforme mostra a figura 1 . Ocorreu leve recuperação após a 24ª semana, mas permaneceu estatisticamente inferior no volume semanal quando comparado a 2019 e 2018 (p<0,01).

**Figura 1:** Volume de atendimentos de unidades de pronto atendimento distribuídos em semanas epidemiológicas (exceto COVID-19)

Seguindo a mudança do perfil dos pacientes observada na tabela 1 , também houve impacto marcante no volume de especialidades médicas no PA, com diminuição estatisticamente significativa (p<0,01) em todas durante o período de estudo, conforme pode ser visto na figura 2 . Notavelmente, a pediatria teve o impacto mais pronunciado, com queda de 70,7% e 71%, em relação a 2019 e 2018, respectivamente. Nos mesmos intervalos, a ortopedia teve redução de 54,6% e 54,2%, a clínica médica de 46,2% e 45,8% e a cirurgia geral de 34,2% e 26,2%.

**Figura 2:** Volume de atendimento conforme especialidades medicas entre os anos

PA: pronto atendimento.

Comparando a proporcionalidade de cada especialidade médica com o volume total, a ortopedia teve ligeira redução, passando de 14,2% nos anos anteriores para 13,6% em 2020, ao passo que a pediatria teve a redução mais pronunciada, com queda de 26,1% e 25,5%, em 2018 e 2019, para 15,8%, em 2020. A clínica médica e a cirurgia geral aumentaram em volume percentual, conforme mostrado na tabela 2 .

**Tabela 2:** Comparação estatística entre o volume de atendimento, conforme especialidades medicas entre os anos

Especialidades médicas	Anos			Valor de p

	2018 n=289.118	2019 n=292.344	2020 n=138.138	
Clínica médica	148.175 (51,2)	149.051 (50,9)	80.251 (58,1)	<0,01
Pediatria	75.499 (26,1)	74.565 (25,5)	21.797 (15,8)	
Ortopedia	41.149 (14,2)	41.553 (14,2)	18.862 (13,6)	
Cirurgia geral	20.534 (7,1)	23.017 (7,9)	15.155 (10,9)	

Resultados expressos por n (%).

O número absoluto de internações hospitalares a partir do PA diminuiu em 31% em 2020, em comparação com 2019, e em 30% em comparação com 2018. No entanto, proporcionalmente, ao se comparar o número mensal de internações hospitalares a partir do PA ao seu respectivo volume total de atendimentos no PA, observou-se aumento estatisticamente significativo (p<0,01) nas taxas de internações a partir de abril de 2020, conforme mostra a figura 3 .

**Figura 3:** Taxas mensais de internação hospitalar a partir de atendimento no pronto atendimento

## DISCUSSÃO

Este estudo mostra evidências de redução de 52% no número de atendimentos no PA no hospital em 2020, em comparação com 2019 e 2018. Embora muito tenha sido postulado sobre as razões para esse fenômeno, como medo de contaminação ou redução da probabilidade de acidentes de trabalho e acidentes de trânsito, ainda não há dados publicados que comprovem tais explicações. Curiosamente, esse número diminuiu de forma acentuada em março de 2020, após a 12ª semana epidemiológica, coincidindo com as medidas de contenção social impostas pelo governo.

Semelhantemente a esta experiência, outras publicações relataram redução nos atendimentos no PA não relacionados à COVID-19, desde março de 2020.^( 11 , 12 )^Dados do *National Syndromic Surveillance Program* (NSSP), nos Estados Unidos, revelaram declínio de 42% nos atendimentos de PA no início da pandemia, de 29 de março a 25 de abril de 2020.^( 10 )^No cenário latino-americano, Pintado et al., mostraram queda de 79,9% no número de pacientes atendidos em um centro de referência para trauma peruano, comparando os meses imediatamente pré e pós- *lockdown* .^( 12 )^

O perfil dos pacientes de acordo com as faixas etárias variou durante a pandemia. Ao contrário da presente expectativa de redução proporcional de idosos, 65% dos pacientes tinham de 60 a 69 anos, em comparação com 56% nos anos anteriores. Montagnon et al., também encontraram aumento semelhante na proporção de pacientes idosos.^( 13 )^No entanto, embora não se conheça o motivo específico disso, talvez seja resultado da redução significativa na proporção de pacientes com menos de 16 anos, que, em 2020, passou de 33% e 22%.

Consoante a essa redução no volume de atendimentos de pacientes mais jovens, também foi encontrada diminuição de 71% nas consultas pediátricas em 2020, em comparação com 2019 e 2018. No início da pandemia, Ciacchini et al., mostraram resultados semelhantes na Itália, com redução de 76% nas internações pediátricas no PA.^( 8 )^Mais recentemente, Nascimento et al., encontraram redução significativa de 81% para 45% nas hospitalizações por doenças respiratórias em crianças menores de 5 anos.^( 14 )^Como outros estudos mostraram o impacto do distanciamento físico e outras estratégias de *lockdown* para desacelerar a propagação de doenças virais respiratórias comuns entre as crianças,^( 15 )^essa redução também poderia ser decorrente de aumento na educação sobre o uso de máscaras e higienização das mãos, tanto em relação a crianças quanto aos pais.

Em relação à gravidade da doença, foi encontrada uma diminuição relativa menor nos casos graves agrupados nos ESI 1 e 2, ao passo que houve diminuição maior no volume de casos leves, agrupados nos ESI 4 e 5. Além disso, estudo alemão realizado por Stöhr et al., encontrou redução nos eventos cardiovasculares eletivos, enquanto as doenças graves e internações inevitáveis, como infarto do miocárdio e acidente vascular cerebral, permaneceram estáveis.^( 16 )^Ainda que demande ser comprovada com dados mais consistentes, essa maior redução nas doenças clínicas leves no PA poderia ser explicada pelo uso disseminado da telessaúde durante a pandemia da COVID-19.^( 17 )^

Apesar de ter havido diminuição de 30% no número absoluto de internações hospitalares provenientes do PA, observa-se aumento proporcional nas taxas de internação desde abril de 2020, como também descrito em outros estudos.^( 18 )^Embora esta análise de dados não o possa comprovar, levanta-se a hipótese de que esse aumento nas taxas de admissão poderia ser decorrente do atraso na procura por cuidados de saúde, resultando em uma apresentação de quadros clínicos mais graves no PA. Suspeita-se que o medo da exposição à infecção da COVID-19 possa ter impedido que fossem administrados cuidados de saúde em tempo hábil e, assim, tenha impactado nos desfechos clínicos.

Este estudo é limitado devido à sua natureza observacional, já que as causas reais da redução observada nos atendimentos do PA não puderam ser determinadas. Além disso, por ser um estudo unicêntrico, não é possível concluir que represente todas as regiões do Brasil, pois o impacto da pandemia da COVID-19 tem variado de acordo com as características dos hospitais e geograficamente em todo o país.

## CONCLUSÃO

A pandemia da COVID-19 resultou em redução significativa no atendimento no pronto atendimento estudado, especialmente nas consultas pediátricas, a partir de março de 2020, logo após as medidas de contenção social impostas pelo governo. Também foi observado aumento proporcional nas taxas de internação hospitalar a partir do pronto atendimento durante o período do estudo. Estes achados sugerem que os profissionais de saúde devem enfatizar a importância dos atendimentos no pronto atendimento para doenças graves, que não podem ser tratadas em outros ambientes.
